# Pemafibrate Improves Lipid and Liver Metabolism in Adult GH Deficiency: A Prospective Observational Study

**DOI:** 10.1210/jendso/bvaf222

**Published:** 2025-12-24

**Authors:** Hiroshi Iesaka, Kyu Yong Cho, So Nagai, Aika Miya, Hiraku Kameda, Hiroshi Nomoto, Akinobu Nakamura, Tatsuya Atsumi

**Affiliations:** Department of Rheumatology, Endocrinology and Nephrology, Faculty of Medicine and Graduate School of Medicine, Hokkaido University, Sapporo, Hokkaido 060-8638, Japan; Department of Rheumatology, Endocrinology and Nephrology, Faculty of Medicine and Graduate School of Medicine, Hokkaido University, Sapporo, Hokkaido 060-8638, Japan; Institute of Health Science Innovation for Medical Care, Hokkaido University, Sapporo, Hokkaido 060-8638, Japan; Department of Rheumatology, Endocrinology and Nephrology, Faculty of Medicine and Graduate School of Medicine, Hokkaido University, Sapporo, Hokkaido 060-8638, Japan; Division of Diabetes and Endocrinology, Department of Medicine, NTT Sapporo Medical Center, Sapporo, Hokkaido 060-0061, Japan; Department of Rheumatology, Endocrinology and Nephrology, Faculty of Medicine and Graduate School of Medicine, Hokkaido University, Sapporo, Hokkaido 060-8638, Japan; Department of Rheumatology, Endocrinology and Nephrology, Faculty of Medicine and Graduate School of Medicine, Hokkaido University, Sapporo, Hokkaido 060-8638, Japan; Department of Rheumatology, Endocrinology and Nephrology, Faculty of Medicine and Graduate School of Medicine, Hokkaido University, Sapporo, Hokkaido 060-8638, Japan; Division of Endocrinology, Metabolism, and Rheumatology, Department of Internal Medicine, Asahikawa Medical University, Asahikawa 078-8510, Japan; Department of Rheumatology, Endocrinology and Nephrology, Faculty of Medicine and Graduate School of Medicine, Hokkaido University, Sapporo, Hokkaido 060-8638, Japan; Department of Rheumatology, Endocrinology and Nephrology, Faculty of Medicine and Graduate School of Medicine, Hokkaido University, Sapporo, Hokkaido 060-8638, Japan

**Keywords:** pemafibrate, adult growth hormone deficiency, dyslipidemia, metabolic dysfunction-associated steatotic liver disease

## Abstract

**Context:**

The outcomes of pemafibrate administration on hypertriglyceridemia and metabolic dysfunction-associated steatotic liver disease associated with adult growth hormone deficiency (AGHD) are unknown.

**Objective:**

To evaluate the effects of pemafibrate on hypertriglyceridemia and liver parameters in AGHD in a real-world setting.

**Design:**

A prospective observational study (December 2019-August 2023).

**Setting:**

Two referral hospitals in Japan.

**Patients:**

Thirty-four consecutive Japanese patients with AGHD complicated with hypertriglyceridemia. Hypertriglyceridemia was defined using a fasting serum triglyceride (TG) concentration of ≥1.7 mmol/L or treated with lipid-lowering medication.

**Treatment:**

Patients were prescribed pemafibrate or continued conventional therapy for 24 weeks.

**Main Outcome Measure:**

The percentage reduction in fasting serum TG level between baseline and 24 weeks was evaluated. Hepatic parameters, including hepatic steatosis index (HSI) derived from aspartate aminotransferase, alanine aminotransferase, and body mass index, were also evaluated, along with other metabolic parameters. The Mann–Whitney U-test and Fisher's exact test were used to compare the change between groups. A multiple regression was performed to identify predictors of TG change.

**Results:**

The change in serum TG level was significantly larger in the pemafibrate group than that in the conventional group (median: −51.0% [interquartile range (IQR): −69.0% to −21.0%] vs −13.0% [IQR: −34.0% to 9.0%], *P* = .0138). HSI decreased after 24 weeks of pemafibrate. Relative TG change correlated with baseline body mass index [regression coefficient (β) .0463, *P* < .0001] and HSI (β .0645, *P* = .0107).

**Conclusion:**

Pemafibrate had beneficial effects on hypertriglyceridemia as well as liver metabolism of patients with AGHD. However, its efficacy was attenuated by obesity.

Adult growth hormone deficiency (AGHD) is characterized by visceral adiposity, low lean body mass, low bone mass, an abnormal lipid profile, and a higher risk of cardiovascular events [1-4]. The gold-standard treatment for AGHD is recombinant human GH replacement, which has been shown to improve body composition and bone metabolism as well as reducing low-density lipoprotein cholesterol concentration [5, 6]. However, GH replacement would not alter serum triglyceride (TG) level [7-10].

The mechanisms of the dysregulation of TG metabolism in AGHD are multifactorial. They include a reduction in visceral fat lipolysis [5, 6], insulin resistance associated with fat accumulation, and lower IGF-1 production secondary to low GH production [11]. In addition, greater hepatic fatty acid uptake and TG synthesis in the liver, secondary to higher CD36 and peroxisome proliferator-activated receptor γ expression [12], de novo hepatic lipogenesis independent of insulin resistance [13], and impaired hepatic free fatty acid β-oxidation [14], have also been reported. GH replacement ameliorates the abnormalities listed here but promotes lipolysis in visceral fat, causing free fatty acids to accumulate in the circulation and liver [15], and suppresses lipoprotein lipase activity [16], which would be expected to offset the effect of GH replacement on TG. Hypertriglyceridemia is a risk factor for cardiovascular events [17], even during statin treatment [18]. AGHD also causes metabolic dysfunction-associated steatotic liver disease (MASLD) through these mechanisms, which is associated with cardiovascular events [19]. Given these links, the treatment of patients with AGHD for hypertriglyceridemia and MASLD is important.

Pemafibrate is a selective peroxisome proliferator-activated receptor α modulator that was approved in 2018 and is a highly selective agonist of human peroxisome proliferator-activated receptor α [20]. It (1) promotes the hepatic β-oxidation of free fatty acids and (2) induces serum TG catabolism by lipoprotein lipase via the inhibition of apolipoprotein CIII, thereby reducing the serum TG concentration [21, 22]. In a previous phase 3 clinical trial, pemafibrate was found to be superior to fenofibrate with respect to the TG-lowering effect and safety [23]. Additionally, pemafibrate was not associated with a higher incidence of adverse events, even when used concomitantly with a statin [24]. The effect of pemafibrate on MASLD has also been reported [25-27]. However, the effects of pemafibrate on hypertriglyceridemia and MASLD associated with AGHD have never been reported. Therefore, it is necessary to determine whether pemafibrate has the same effect in patients with AGHD as in those with hypertriglyceridemia of other etiologies. This has not been evaluated to date because of the rarity of the disease. The purpose of the present study was to evaluate the effects of pemafibrate on the hypertriglyceridemia and liver parameters of patients with AGHD in a real-world clinical setting.

## Materials and Methods

### Study Sample

The eligible patients were ≥20 years of age and had AGHD and hypertriglyceridemia. Patients with AGHD had been tested according to the Japanese guidelines regarding hypothalamo-pituitary dysfunction before the diagnosis [28]. GH stimulation tests, such as the GH-releasing peptide-2 test [29] and/or insulin tolerance test, had been performed [30] in patients with pituitary disease. Hypertriglyceridemia was defined using a fasting TG concentration of ≥1.7 mmol/L [31]. Patients taking TG-lowering medications containing other fibrates, such as fenofibrate and bezafibrate, were also included. Both those who were undergoing GH replacement and those who were not were included. The exclusion criteria were as follows: pregnancy, severe liver dysfunction (Child–Pugh class B or C), current gallstones, and lack of suitability for the study for other reasons, as determined by the attending physician. The patients were recruited at the NTT Sapporo Medical Center and Hokkaido University Hospital in Hokkaido, Japan.

### Study Design

We performed a 2-center, prospective, observational study between December 2019 and August 2023. The pemafibrate group comprised fibrate-naïve patients who initiated pemafibrate therapy and those who were switched from another fibrate to pemafibrate. Pemafibrate was started at 0.1 mg twice a day (0.2 mg/day) but could be increased to 0.2 mg twice a day (0.4 mg/day) at the discretion of the attending physician if the patient's fasting TG concentration was ≥1.7 mmol/L [31]. The conventional group included patients who were not taking any medication for hypertriglyceridemia or who continued to take another fibrate. All the participants were encouraged to maintain the same dietary and exercise habits throughout the study. Those who were diagnosed with hypertriglyceridemia with AGHD were enrolled when the TG level at observation initiation was normal, because they had received conventional fibrates.

Clinical and laboratory data, including metabolic parameters such as the circulating TG concentration, the level of other lipids, liver parameters, platelet count (PLT), albumin concentration, IGF-1 concentration, plasma glucose concentration, and immunoreactive insulin were measured after an overnight fast at baseline and after 24 weeks of treatment. The body mass, blood pressure, and waist circumference of each participant were also measured at baseline and at week 24. To evaluate steatosis, the hepatic steatosis index (HSI) [32] was calculated. Participants with an HSI ≥ 30, except for those with an alcohol intake greater than that permissible for MASLD (>30 g/day for men and > 20 g/day for women), viral hepatitis, autoimmune hepatitis, or primary biliary cholangitis were considered to have MASLD [33-35]. Fatty liver index was not evaluated because TG is included in the formula used to calculate this index. Markers of hepatic fibrosis, such as the Fibrosis-4 index (FIB-4) [36] and the nonalcoholic fatty liver disease fibrosis score (NFS) [37], were also evaluated at baseline and 24 weeks. HSI, FIB-4, and NFS were calculated using the following formulae:

HSI = 8 × [alanine aminotransferase (ALT)/aspartate aminotransferase (AST) ratio] + body mass index (BMI) (kg/m^2^) (+2, if diabetes; +2, if female)

FIB-4 = age (years) × AST (U/L)/[PLT (10^9^/L) × [ALT(U/L)]^1/2^]

NFS = −1.675 + 0.037 × age (years) + 0.094 × BMI (kg/m^2^) + 1.13 × IFG/diabetes (yes = 1, no = 0) + 0.99 × (AST/ALT ratio) − 0.013 × PLT (10^9^/L) − 0.66 × albumin (g/dL)

During the study period, 2 methods were used to measure IGF-1: an immunoradiometric assay kit (IRMA) (IGF-1 assay; Fuji Rebio Inc., Tokyo, Japan) and an electrochemiluminescence immunoassay (ECLIA) (Elecsys® IGF-1, Roche Diagnostics K.K., Tokyo, Japan). The IGF-1 concentrations measured using IRMA and ECLIA closely correlated [r = 0.994; IGF-1 (ECLIA) = 1.022 × IGF-1 (IRMA) − 7.230, according to data of SRL, Inc.]. The method used to measure ALP activity was changed from the Japan Society of Clinical Chemistry (JSCC) method to the International Federation of Clinical Chemistry and Laboratory Medicine (IFCC) method in Japan, and during the present study, the ALP activity was measured using the IFCC method or calculated as follows: IFCC value = JSCC value × 0.335 + 0.995 [38].

### Primary and Secondary Endpoints

The primary endpoint of the study was the percentage change in the fasting serum TG concentration between baseline and 24 weeks. The secondary endpoints included the percentage changes in other lipid parameters; in liver-related parameters, including platelet count and albumin concentration; in the scoring systems HSI, FIB-4, and NFS; and in Homeostasis Model Assessment 2 for Insulin Resistance, BMI, waist circumference, and blood pressure. Changes in lipid concentrations are expressed as percentage changes because these are recommended for evaluating the efficacy of lipid-lowering agents in guidelines and have been used in clinical trials [39, 40]. The factors associated with the changes in TG were also researched.

### Ethics Approval

The study protocol was approved by the Institutional Review Board of NTT Sapporo Medical Center (19-00588) prior to commencing the study. Additional matters requiring approval, such as protocol amendments, were reviewed and approved by the institutional review boards as required. The study was conducted in accordance with the principles of the Declaration of Helsinki. All the participants provided their written informed consent prior to participation. The study was registered in the University Hospital Medical Information Network Clinical Trials Registry (UMIN000038558).

### Statistical Analysis

The results are expressed as median (first quartile to third quartile). Differences in the baseline characteristics of the groups were compared using the chi-square test or Fisher's exact test. The Wilcoxon test was used for pre- and posttreatment comparison, and the Mann–Whitney U-test (Wilcoxon rank-sum test) was used for comparisons of changes in parameters between the groups. Multiple regression analysis was used to evaluate the relationships between TG and other parameters. Two-sided *P*-values of < .05 were considered to be statistically significant. Statistical analyses were performed using JMP Statistical Database Software version 17.0.0 (SAS Institute, Inc., Cary, NC, USA).

AGHD is a relatively rare disease, and it was anticipated that it would be difficult to specify the number of cases required in advance. Therefore, a post hoc analysis was conducted to evaluate the statistical power of the evaluations made.

## Results

### Baseline Characteristics of the Participants

**Figure 1.**
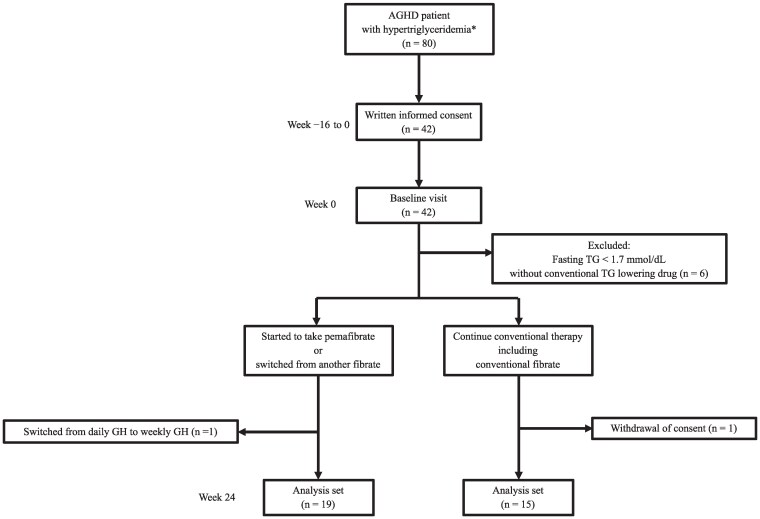
Flow diagram for the study. * Hypertriglyceridemia: fasting TG ≥ 1.7 mmol/L or postprandial TG ≥ 2.0 mmol/L. Abbreviations: AGHD, adult growth hormone disease; TG, triglyceride.

Eighty patients were screened, and written informed consent was obtained from 42. Of these, 36 met the inclusion criteria and were enrolled, but 2 dropped out for the reasons shown in Fig. 1. Therefore, data from 34 participants were analyzed: 19 in the pemafibrate group and 15 in the conventional group. Three patients in the pemafibrate group and 2 in the conventional group had previously taken another fibrate. The dose of pemafibrate administered was 0.1 mg twice a day (0.2 mg/day) for all the participants in the pemafibrate group throughout the study. The baseline characteristics of the participants are shown in Table 1. The baseline clinical and laboratory parameters did not significantly differ between the groups, except for the high-density lipoprotein-cholesterol concentration (Table 1). During the study period, there were no serious adverse events necessitating withdrawal from the study in either group.

**Table 1.** Demographic and clinical characteristics of the participants at baseline

Variable	Pemafibrate group (n = 19)	Conventional treatment group (n = 15)	*P*-value
Age (years)	59.0 (41.0-72.0)	56.0 (46.0-69.0)	.9447
Female sex, n (%)	7 (36.8)	7 (46.7)	.7282
BMI (kg/m^2^)	25.9 (22.7-27.6)	27.1 (24.3-29.4)	.4250
Waist circumference (cm)	87.0 (84.5-99.5)	93.0 (86.0-101.0)	.7478
Systolic blood pressure (mmHg)	130.0 (114.0-138.0)	130.0 (120.0-146.0)	.8080
Diastolic blood pressure (mmHg)	81.0 (74.0-89.0)	83.0 (68.0-86.0)	.9308
Duration of AGHD (years)	4.8 (2.8-9.3)	9.7 (5.2-12.4)	.0660
Original pituitary disease, n (%)			
Pituitary neuroendocrine tumor	8 (42.1)	7 (46.7)	1.0000
Craniopharyngioma	5 (26.3)	3 (20.0)	1.0000
Others	6 (31.6)	5 (33.3)	1.0000
MASLD	13 (68.4)	13 (86.7)	.2569
Medication			
Statin, n (%)	8 (42.1)	10 (66.7)	.1854
Hydrocortisone replacement, n (%)	10 (52.6)	8 (53.3)	1.0000
Levothyroxine replacement, n (%)	16 (84.2)	9 (60.0)	.1392
Gonadal replacement, n (%)	12 (63.1)	6 (40.0)	.2998
GH replacement, n (%)	13 (68.4)	9 (60.0)	.7241
T-chol (mmol/L)	4.8 (4.3-5.7)	5.2 (4.4-6.1)	.5209
TG (mmol/L)	2.4 (1.8-3.0)	2.3 (1.9-2.8)	.9171
HDL-C (mmol/L)	1.1 (0.9-1.3)	1.4 (1.2-1.6)	.0119*
LDL-C (mmol/L)	2.6 (2.3-3.1)	2.8 (2.4-3.6)	.4020
Fasting glucose (mmol/L)	5.6 (5.4-6.8)	5.4 (5.1-6.6)	.4556
Fasting IRI (pmol/L)	63.5 (41.4-81.6)	78.2 (56.7-109.8)	.1334
HbA1c (mmol/mol)	38.8 (35.5-46.5)	38.8 (35.5-43.2)	.8345
Diabetes, n, (%)	3 (15.8)	2 (13.3)	1.0000

Abbreviations: AGHD, adult growth hormone deficiency; BMI, body mass index; HbA1c, hemoglobin A1c; HDL, high-density lipoprotein-cholesterol; IRI, immunoreactive insulin; LDL-C, low-density lipoprotein-cholesterol; MASLD, metabolic dysfunction-associated steatotic liver disease; T-chol, total cholesterol; TG, triglyceride.

Values are expressed as median (interquartile range) or number (%). Differences between the groups were assessed using the Mann–Whitney U-test or Fisher's exact test.

* *P* < .05, ** *P* < .01.

### Changes in Lipid and Liver-related Parameters

The primary efficacy analysis demonstrated a significantly larger percentage reduction in serum TG levels in the pemafibrate group than in the conventional group (Table 2). There was no change in the low-density lipoprotein cholesterol or high-density lipoprotein cholesterol concentration in either group. The reduction in ALP activity was significantly larger in the pemafibrate group than in the conventional group, but the changes in the AST and ALT activities did not differ between the groups (Table 2). The gamma-glutamyl transferase (γ-GTP), HSI, NFS, IGF-1 SD score (SDS), fasting plasma glucose, and Homeostasis Model Assessment 2 for Insulin Resistance decreased and the platelet count increased significantly from baseline in the pemafibrate group, but there were no significant differences in these changes from the conventional group (Table 2).

**Table 2.** Changes from baseline in the primary endpoint and key secondary efficacy endpoints

	Pemafibrate group (n = 19)		Conventional treatment group (n = 15)		*P-*value for the difference in the change between the groups
Vvariable	Baseline	Mean change over 24 weeks	Baseline	Mean change over 24 weeks	
Δ%Triglyceride (mmol/L)	2.4 (1.8 to 3.0)	−51.0% (−69.0 to −21.0) **	2.3 (1.9 to 2.8)	−13.0% (−34.0 to 9.0)	.0138†
Δ%HDL-C (mmol/L)	1.1 (0.9 to 1.3)	9.0% (2.0 to 27.0)	1.4 (1.2 to 1.6)	5.0% (−2.0 to 8.0)	.2823
Δ%LDL-C (mmol/L)	2.6 (2.3 to 3.1)	3.0% (−8.0 to 28.0)	2.8 (2.4 to 3.6)	−4.0% (−11.0 to 5.0)	.2365
AST (U/L)	25.0 (20.0 to 33.0)	1.0 (−4.0 to 5.0)	27.0 (20.0 to 34.0)	−3.0 (−9.0 to 4.0)	.1982
ALT (U/L)	25.0 (16.0 to 35.0)	−1.0 (−8.0 to 3.0)	27.0 (16.0 to 34.0)	−3.0 (−9.0 to 0.0)	.5784
γ-GTP (U/L)	30.0 (21.0 to 76.0)	−10.0 (−17.0 to −4.0) **	33.0 (21.0 to 68.0)	−3.0 (−20.0 to 0.0) *	.1446
ALP (U/L)	74.0 (51.0 to 99.0)	−22.0 (−39.6 to −15.0) **	74.0 (66.0 to 83.0)	−5.0 (−7.0 to −2.0)	<.0001††
PLT (/10^9)	241 (212 to 277)	20 (13 to 46) **	254 (203 to 273)	14 (−12 to 24)	.1499
ALB (g/L)	43.0 (42.0 to 46.0)	2.0 (0.0 to 4.0) **	42.0 (41.0 to 44.0)	1.0 (−2.0 to 2.0)	.0756
HSI	33.7 (30.4 to 41.9)	−0.8 (−2.7 to −0.6) **	33.2 (31.7 to 40.3)	−1.2 (−2.1 to 1.2)	.4052
NFS	−1.648 (−2.263 to −0.424)	−0.331 (−0.669 to 0.312)*	−1.091 (−1.736 to −0.659)	−0.097 (−0.343 to 0.225)	.3145
FIB-4 index	1.22 (0.86 to 1.48)	0.01 (−0.10 to 0.10)	1.36 (0.80 to 1.94)	−0.08 (−0.29 to 0.33)	.3315
IGF-1 SDS	−0.03 (−2.70 to 0.68)	−0.33 (−0.98 to 0.00)*	−0.03 (−2.51 to 0.85)	−0.25 (−1.17 to 0.32)	.9309
Fasting plasma glucose (mmol/L)	5.6 (5.4 to 6.8)	−0.2 (−0.8 to 0.3)*	5.4 (5.1 to 6.6)	0.0 (−0.3 to 0.2)	.3575
Fasting IRI (pmol/L)	63.5 (41.4 to 81.6)	−8.6 (−21.2 to −0.5)	78.2 (56.7 to 109.8)	−9.3 (−56.0 to 17.9)	.9280
HOMA-2R	1.36 (0.92 to 1.93)	−0.19 (−0.47 to −0.04)*	1.81 (1.25 to 2.35)	−0.20 (−1.32 to 0.40)	.9567
BMI (kg/m^2^)	25.9 (22.7 to 27.6)	0.1 (−0.6 to 0.3)	27.1 (24.3 to 29.4)	0.0 (−0.7 to 0.4)	.7024
Waist circumference (cm)	87.0 (84.5 to 99.5)	0.0 (−1.8 to 4.5)	93.0 (86.0 to 101.0)	−0.8 (−3.0 to 5.0)	.8353
Systolic blood pressure (mmHg)	130.0 (114.0 to 138.0)	−3.0 (−11.0 to 10.0)	130.0 (120.0 to 146.0)	−2.0 (−15.0 to 11.0)	1.0000

Abbreviations: ALB, albumin; ALP, alkaline phosphatase; ALT, alanine aminotransferase; AST, aspartate aminotransferase; BMI, body mass index; γ-GTP, gamma-glutamyl transferase; HDL-C, high-density lipoprotein-cholesterol; HOMA-2R, Homeostasis Model Assessment 2 for Insulin Resistance; HSI, hepatic steatosis index; IGF-1 SDS, insulin-like growth factor 1 SD score; IRI, immunoreactive insulin; LDL-C, low-density lipoprotein-cholesterol; NFS, nonalcoholic fatty liver disease fibrosis score; PLT, platelet count; TG, triglyceride.

Changes in lipid parameters are expressed as percentage changes from baseline. * *P* < .05, ** *P* < .01 vs baseline. †*P* < .05, ††*P* < .01 vs the conventional group.

### Subgroup Analysis

Subsequently, the effects of pemafibrate were evaluated under specific conditions. Among the participants undergoing GH supplementation, there was a tendency toward a reduction in TG concentration in the pemafibrate group, but this was not statistically significant. There was no significant difference in the IGF-1 SDS in the subgroup that was undergoing GH supplementation (Table 3), but in the subgroup that was not, there was a percentage change in TG concentration of −21.0% [−77.0% to −2.0%] associated with pemafibrate administration. In the absence of GH supplementation, the difference in IGF-1 SDS from baseline was −0.61 [−1.47 to −0.24] (*P* = .0313). In the participants with MASLD, the reduction in the TG concentration was smaller, and there were no statistically significant differences in the changes between the 2 groups. Conversely, the γ-GTP and HSI of the pemafibrate group decreased similarly to those being treated with pemafibrate in the entire cohort (Table 4).

**Table 3.** Changes from baseline in key parameters in the GH replacement subgroup

	Pemafibrate group (n = 13)		Conventional treatment group (n = 9)		*P*-value for the difference in the change between the groups
Variable	Baseline	Mean change over 24 weeks	Baseline	Mean change over 24 weeks	
Δ% Triglyceride (mmol/L)	2.4 (2.0 to 2.8)	−53.0% (−65.0 to −36.0)**	2.5 (2.2 to 3.2)	−23.0% (−41.0 to −2.0)*	.0528
Δ% HDL-C (mmol/L)	1.1 (0.9 to 1.4)	16.0% (−6.0 to 27.0)	1.4 (1.2 to 1.7)	4.0% (−3.0 to 11.0)	.3853
Δ% LDL-C (mmol/L)	2.9 (2.5 to 3.4)	2.0% (−9.0 to 17.0)	3.1 (2.5 to 4.0)	0.0% (−11.0 to 8.0)	.7894
AST (U/L)	22.0 (19.5 to 26.5)	1.0 (−4.0 to 4.5)	27.0 (19.0 to 31.0)	−3.0 (−10.5 to 7.0)	.3662
ALT (U/L)	25.0 (15.5 to 34.0)	−1.0 (−11.5 to 3.0)	24.0 (16.5 to 33.0)	−3.0 (−10.0 to 3.5)	1.0000
γ-GTP (U/L)	30.0 (18.0 to 44.0)	−10.0 (−19.0 to −4.0)**	33.0 (18.0 to 66.5)	−3.0 (−21.5, 0.0)	.4613
ALP (U/L)	74.0 (50.5 to 95.0)	−17.0 (−39.7 to −12.3)**	77.0 (70.0 to 86.5)	−6.0 (−8.5 to 5.0)	.0005††
PLT (/10^9^)	267 (217 to 321)	21 (8 to 46)**	267 (208 to 300)	23 (10 to 31)*	.6163
ALB (g/L)	44.0 (42.0 to 46.0)	3.0 (1.0 to 4.0)**	42.0 (40.5 to 44.0)	0.0 (−1.5 to 2.5)	.0514
HSI	32.3 (29.4 to 40.3)	−0.7 (−2.4 to −0.4)*	33.2 (31.6 to 40.7)	−1.2 (−2.2 to 1.5)	.9468
NFS	−1.725 (−2.573 to −1.223)	−0.373 (−0.675 to 0.106)*	−1.148 (−2.397 to −0.740)	−0.204 (−0.439 to −0.007)*	.5043
FIB-4 index	1.08 (0.85 to 1.34)	0.01 (−0.08 to 0.13)	1.28 (0.63 to 1.61)	−0.13 (−0.24 to −0.01)	.0384†
IGF-1 SDS	0.62 (−0.04 to 0.83)	−0.14 (−0.94 to 0.08)	0.51 (−1.25 to 1.31)	−0.29 (−1.21 to 0.39)	.5930
Fasting plasma glucose (mmol/L)	5.6 (5.1 to 6.7)	−0.3 (−0.9 to 0.3)*	5.3 (5.2 to 6.4)	0.0 (−0.4 to 0.5)	.4032
Fasting IRI (pmol/L)	55.2 (39.8 to 71.8)	−7.2 (−18.3 to 0.4)	78.2 (52.4 to 98.7)	−9.3 (−44.5 to 19.7)	.8937
HOMA-2R	1.32 (0.89 o 1.63)	−0.16 (−0.41 to −0.01)*	1.81 (1.13 to 2.20)	−0.20 (−1.02 to 0.47)	.9468
BMI (kg/m^2^)	23.6 (22.5 to 27.1)	0.1 (−0.5 to 0.4)	27.7 (23.4 to 31.3)	0.0 (−0.9 to 0.6)	.7632
Waist circumference (cm)	86.0 (82.0 to 93.0)	3.0 (−1.5 to 5.0)	93.0 (82.0 to 100.0)	0.0 (−2.1 to 3.3)	.4694
Systolic blood pressure (mmHg)	132.0 (118.0 to 143.5)	−4.0 (−15.0 to 6.0)	126.0 (117.0 to 139.0)	3.0 (−15.5 to 13.5)	.4621

Abbreviations: ALB, albumin; ALP, alkaline phosphatase; ALT, alanine aminotransferase; AST, aspartate aminotransferase; BMI, body mass index; GH, growth hormone; γ-GTP, gamma-glutamyl transferase; HDL-C, high-density lipoprotein-cholesterol; HOMA-2R, Homeostasis Model Assessment 2 for Insulin Resistance; HSI, hepatic steatosis index; IGF-1 SDS, insulin-like growth factor 1 SD score; IRI, immunoreactive insulin; LDL-C, low-density lipoprotein-cholesterol; NFS, nonalcoholic fatty liver disease fibrosis score; PLT, platelet count; TG, triglyceride.

Changes in lipid parameters are expressed as percentage changes from baseline. * *P* < .05, ** *P* < .01 vs baseline. †*P* < .05, ††*P* < .01 vs the conventional group.

**Table 4.** Changes from baseline in key parameters in the MASLD subgroup

	Pemafibrate group (n = 13)		Conventional treatment group (n = 13)		*P*-value for the difference in the change between the groups
Variable	Baseline	Mean change over 24 weeks	Baseline	Mean change over 24 weeks	
Δ% Triglyceride (mmol/L)	2.1 (1.7 to 2.9)	−23.0% (−63.0 to −3.0)**	2.3 (2.0 to 2.9)	−23.0% (−34.0 to 8.0)	.2815
Δ% HDL-C (mmol/L)	1.1 (0.9 to 1.1)	7.0% (2.0 to 24.0)	1.3 (1.2 to 1.5)	6.0% (0.0 to 11.0)	.6444
Δ% LDL-C (mmol/L)	2.6 (2.4 to 3.0)	2.0% (−9.0 to 32.0)	2.7 (2.4 to 3.4)	−1.0% (−6.0 to 6.0)	.7237
AST (U/L)	25.0 (19.5 to 31.5)	0.0 (−4.0 to 6.5)	27.0 (20.5 to 35.5)	−3.0 (−11.0 to 5.0)	.3415
ALT (U/L)	25.0 (16.5 to 35.0)	−1.0 (−7.5 to 3.5)	28.0 (21.5 to 35.0)	−4.0 (−9.5 to 1.0)	.4257
γ-GTP (U/L)	30.0 (22.0 to 78.5)	−10.0 (−20.0 to −4.0)**	59.0 (25.0 to 78.0)	−3.0 (−25.0 to 1.5)	.2371
ALP (U/L)	74.0 (55.5 to 100.5)	−23.0 (−39.0 to −13.8)**	74.0 (67.5 to 86.5)	−5.0 (−7.0 to 1.5)	<.0001††
PLT (/10^9)	248 (211 to 321)	19 (7 to 39)	249 (202 to 276)	14 (−7 to 26)*	.5212
ALB (g/L)	43.0 (43.0 to 46.0)	1.0 (−0.5 to 3.0)*	43.0 (40.5 to 44.0)	1.0 (0.0 to 2.5)	.6237
HSI	36.3 (32.3 to 42.6)	−0.7 (−2.3 to −0.4)*	35.9 (32.7 to 40.7)	−1.2 (−2.2 to 0.5)	.8375
NFS	−1.648 (−2.526 to −0.479)	−0.125 (−0.503 to 0.333)	−1.091 (−1.663 to −0.583)	−0.097 (−0.282 to 0.169)	.9183
FIB-4 index	1.01 (0.60 to 1.44)	0.04 (−0.05 to 0.08)	1.36 (0.83 to 1.79)	−0.08 (−0.45 to 0.22)	.2382
IGF-1 SDS	−1.00 (−3.52 to 0.73)	−0.51 (−0.935 to −0.135)	0.05 (−2.05 to 0.87)	−0.29 (−1.17 to 0.09)	.9591
Fasting plasma glucose (mmol/L)	5.7 (5.4 to 7.6)	−0.3 (−0.8 to 0.1)	5.5 (5.3 to 6.6)	0.0 (−0.4 to 0.2)	.3554
Fasting IRI (pmol/L)	72.5 (54.0 to 94.5)	−11.8 (−40.0 to −2.5)	89.7 (51.3 to 127.4)	−9.3 (−57.0 to 27.6)	.6438
HOMA-2R	1.68 (1.33 to 2.06)	−0.28 (−0.96 to −0.12)	2.04 (1.13 to 2.73)	−0.20 (−1.33 to 0.64)	.6833
BMI (kg/m^2^)	27.2 (24.7 to 31.7)	0.2 (−0.5 to 0.4)	27.7 (24.4 to 31.0)	0.0 (−0.7 to 0.4)	.3688
Waist circumference (cm)	94.0 (87.0 to 104.8)	0.3 (−1.9 to 4.8)	95.4 (86.5 to 101.0)	−0.8 (−2.1 to 3.3)	.7439
Systolic blood pressure (mmHg)	130.0 (116.0 to 134.5)	0.0 (−10.0 to 11.0)	130.0 (122.0 to 141.0)	−2.0 (−15.5 to 7.0)	.4720

Abbreviations: ALB, albumin; ALP, alkaline phosphatase; ALT, alanine aminotransferase; AST, aspartate aminotransferase; BMI, body mass index; γ-GTP, gamma-glutamyl transferase; HDL-C, high-density lipoprotein-cholesterol; HOMA-2R, Homeostasis Model Assessment 2 for Insulin Resistance; HSI, hepatic steatosis index; IGF-1 SDS, insulin-like growth factor 1 SD score; IRI, immunoreactive insulin; LDL-C, low-density lipoprotein-cholesterol; MASLD, metabolic dysfunction-associated steatotic liver disease; NFS, nonalcoholic fatty liver disease fibrosis score; PLT, platelet count; TG, triglyceride.

Changes in lipid parameters are expressed as percentage changes from baseline. * *P* < .05, ** *P* < .01 vs baseline. †*P* < .05, ††*P* < .01 vs the conventional group.

### Correlation Analysis

In addition to the reduction in TG, there were improvements in some of the other parameters, with some being statistically significant. We performed univariate analysis of these parameters for the participants in the pemafibrate group (n = 19). The variables were selected based on all baseline values, a significant difference in the change between the groups, and/or a significant change during the study. The baseline BMI and baseline HSI positively correlated with the change in TG concentration. Age and female sex were negatively associated, and gonadotropin replacement was positively associated, with the change in TG. The change in HSI tended to positively correlate with the change in TG, but this was not significant (Table 5).

**Table 5.** Relationships between the change in TG and other parameters in the pemafibrate group

Baseline	Correlation coefficient	*P*	Variable	Correlation coefficient	*P*
Age	−0.5217	.0220*			
Sex	—	.0046			
Hydrocortisone	—	.4877			
Levothyroxine	—	.7799			
Gonadotropin	—	.0160*			
GH replacement	—	.5107			
HDL-C (mmol/L)	−0.0062	.9801			
LDL-C (mmol/L)	0.2089	.3908			
AST (U/L)	0.0879	.7206			
ALT (U/L)	0.3553	.1355			
γ-GTP (U/L)	0.3443	.1488	Δγ-GTP (U/L)	−0.1789	.4636
ALP (U/L)	−0.0333	.8922	ΔALP (U/L)	0.1694	.4882
PLT (/10^9)	0.0728	.7670	ΔPLT (/10^9)	−0.2678	.2677
ALB (g/L)	0.3137	.1910	ΔALB (g/L)	−0.2538	.2945
HSI	0.7140	.0006**	ΔHSI	0.4439	.0570
NFS	−0.3351	.1608	ΔNFS	0.2772	.2506
FIB-4 index	−0.3526	.1387			
IGF-1 SDS	−0.3589	.1313	ΔIGF-1 SDS	0.3396	.1549
Fasting plasma glucose (mmol/L)	−0.1537	.5298	ΔFasting plasma glucose (mmol/L)	0.1080	.6598
Fasting IRI (pmol/L)	0.4448	.0644			
HOMA-2R	0.4076	.0931	ΔHOMA-2R	0.1496	.5534
BMI (kg/m^2^)	0.7117	.0006**			
Waist circumference (cm)	0.4757	.0536			
Systolic blood pressure (mmHg)	−0.1493	.5418			

Abbreviations: ALB, albumin; ALP, alkaline phosphatase; ALT, alanine aminotransferase; AST, aspartate aminotransferase; BMI, body mass index; GH, growth hormone; γ-GTP, gamma-glutamyl transferase; HDL-C, high-density lipoprotein-cholesterol; HOMA-2R, Homeostasis Model Assessment 2 for Insulin Resistance; HSI, hepatic steatosis index; IGF-1 SDS, insulin-like growth factor 1 SD score; IRI, immunoreactive insulin; LDL-C, low-density lipoprotein-cholesterol; NFS, nonalcoholic fatty liver disease fibrosis score; PLT, platelet count; TG, triglyceride.

Spearman's rank test was used. * *P* < .05, ** *P* < .01.

Next, we performed an adjusted analysis of the relationships between the percentage reductions in TG and other parameters, using the variables that were significant in the unadjusted analysis, such as age, sex, and baseline BMI. We confirmed there was no multicollinearity by calculating the variance inflation factor among the parameters we used in the adjusted analysis. Baseline BMI was identified as a significant predictor of this change, but because the baseline HSI showed collinearity with the baseline BMI (variance inflation factor 6.7) and includes baseline BMI, it was excluded from the analysis. Although the change in HSI was not significant in the unadjusted analysis, liver parameters are clinically important and related to the TG concentration [26-28], and therefore we included it in the analysis. By the stepwise selection of variables according to their *P*-values on unadjusted analysis, we identified the baseline BMI and the change in HSI as independent and significant predictors of a decrease in TG (Table 6). To evaluate the stability of regression coefficients, bootstrap resampling with 1000 iterations was performed. During each resampling, regression models were reconstructed using the baseline BMI and delta HSI as independent variables. The distribution of coefficients is now summarized using the bootstrap mean, SD, and 95% confidence interval.

**Table 6.** Relationships between clinically relevant parameters and the change in TG, according to multiple regression analysis

	Unadjusted analysis		Adjusted analysis		
Factor	Correlation coefficient	*P*-value	Regression coefficient	95% confidence interval	*P*-value
Age (years)	−0.5217	.0220			
Sex	—	.0046			
BMI (kg/m^2^)	0.7117	.0006	0.0463	0.0291 to 0.0636	<.0001**
delta HSI	0.4439	.0570	0.0645	0.0172 to 0.1119	.0107*

Abbreviations: BMI, body mass index; HSI, hepatic steatosis index; TG, triglyceride.

Stepwise multiple regression analysis was performed. * *P* < .05, ** *P* < .01.

### Review of the Sample Size and Clinical Significance of the Findings

A post hoc power analysis was performed using α = .05, which yielded a power of 0.7930. In addition, Hedge's *g* was calculated to measure the size of the clinical effect, and it was 1.21. A 38.0% reduction in TG concentration and estimated SDs of 32.6% for the pemafibrate group and 31.9% for the conventional group were consistent with the results of some previous phase 2 or 3 pemafibrate trials, which demonstrated 33.5% to 71.1% reductions, with SDs of 18.3% to 40.2% [40-43]. Although the sample size in the present study was quite small, these results suggest that the findings have clinical significance.

## Discussion

In the present study, we have demonstrated reductions in the circulating TG concentrations of patients with AGHD as a result of pemafibrate therapy, along with decreases in HSI. We identified a positive correlation between the reductions in HSI and TG. Furthermore, there were complementary changes in some liver-related and other parameters in association with pemafibrate treatment.

Several mechanisms have been proposed to contribute to the dyslipidemia in patients with AGHD, and there has been concern that the effect of pemafibrate in this group may be less marked than in patients with hypertriglyceridemia of other etiologies. The present study provides evidence that pemafibrate is an effective means of reducing the serum TG concentrations of patients with AGHD. The magnitude of the reduction in TG was comparable to that identified in previous studies of patients without AGHD [39-43]. In patients who underwent GH supplementation, the reduction in TG followed a similar trend but was not significant, owing to the small sample size. Furthermore, the identified decrease in TG concentration occurred alongside the reduction in HSI and was more pronounced in the participants with lower baseline BMIs.

We also identified significant changes in HSI, NFS, γ-GTP, albumin, and fasting plasma glucose within the pemafibrate group, similar to those identified in the general population [28, 39, 41-43]. The negative findings for AST and ALT may be explained by the low baseline ALT [44]. A 1-year treatment duration is necessary for the induction of changes in markers of liver fibrosis [45], and therefore long-term studies should be performed to confirm the effects of pemafibrate on the liver fibrosis of patients with AGHD.

The effect of pemafibrate on TG concentration is attenuated in patients with obesity, and indeed, the effect of pemafibrate on MASLD is larger in lean patients [46]. Therefore, pemafibrate could have a similar effect on the TG levels in patients with AGHD and a high BMI. It has also been suggested that weight management is important in people with AGHD who are undergoing GH replacement therapy [47]. Taken together, these findings suggest that weight management is important prior to medical therapy. In the present study, the dose of pemafibrate administered was not increased for this group, so it remains to be determined whether a higher dose would have the same TG-lowering effect as in lean patients.

There was no clear linear relationship between the level of GH replacement therapy and the size of the change in TG concentration. However, the reduction in TG was attenuated and the IGF-1 SDS significantly decreased in the subgroup that was not undergoing GH replacement. Therefore, with respect to TG metabolism, regular monitoring of IGF-1 SDS and an appropriate level of GH replacement are important.

The principal finding of the present study was that pemafibrate has a TG-lowering effect in patients with AGHD, and this was accompanied by a correlation between the reduction in triglyceridemia and the amelioration of hepatic steatosis. Thus, pemafibrate is not only safe but may also be beneficial for hepatic lipid metabolism and be useful for the management of MASLD. However, the study had the following limitations. (1) It was an observational study; therefore, the possibility of unmeasured confounding cannot be excluded. (2) The study was small; therefore, few subanalyses were performed. (3) The appropriate diet and exercise level of the participants were not defined in the study protocol and were not evaluated during the study. However, the diet and exercise recommendations in the guidelines of the Japan Atherosclerosis Society [31] were adopted by the endocrinologists, and the participants showed minimal changes in body mass. (4) The effects of dose escalation were not evaluated. (5) The fibrosis scores did not show differing changes between the groups, possibly because of the short duration of the study.

In the future, the clinical course of hypertriglyceridemia and MASLD in pemafibrate-treated patients with AGHD should be evaluated using a larger sample. Specifically, a 2-pronged approach would be important: (1) to investigate the effects of GH supplementation status, the primary disease and the presence of other endocrine disorders or supplementation through subgroup analyses and (2) to monitor the course of hypertriglyceridemia and MASLD over a longer period to better understand their relationship and the sustained effects of pemafibrate in patients with AGHD.

In conclusion, pemafibrate had beneficial effects on the hypertriglyceridemia and liver metabolism of patients with AGHD, although its efficacy was attenuated by obesity.

## Data Availability

The datasets collected during the present study will be made available by the corresponding author on reasonable request.

## Clinical Trial Information

Clinical trial registration: UMIN000038558
